# Supplementary material for: Improved Anther Culture Media for Enhanced Callus Formation and Plant Regeneration in Rice (Oryza sativa L.)
Source: Plants (Basel). 2021 Apr 22;10(5):839. doi: 10.3390/plants10050839 (PMC8143452; doi:10.3390/plants10050839)
Supplement: Supplementary file 1 [file plants-10-00839-s001.zip › plants-1138235-supplementary.pdf]

Supplementary Table 1 Results of Analysis of variance (ANOVA) for the percentage of green plantlet regeneration (RGP), percentage of albino plants regeneration (PAP), regeneration efficiency of green plantlets (RGPE), and regeneration efficiency of albino plantlets (PAPE).

	Df	MS of RGP	MS of PAP	MS of RGPE	MS of PAPE
**Genotype**	2	21578***	5379***	2193***	302.70***
**Media**	3	14628***	3186***	4124***	219.80***
**Genotype : Media**	6	2342***	3196***	628***	90.35***
**Residuals**	48	5	13	20	92

Data were statistically analyzed with two-way ANOVA (P=0.05).

*** Significant at the 0.001 probability level.
